# Characteristics and outcomes of hospitalized patients with Isolated and systemic cardiac sarcoidosis: Analysis of the Nationwide readmissions database 2016–2021

**DOI:** 10.1016/j.ijcha.2025.101636

**Published:** 2025-02-24

**Authors:** Raheel Ahmed, Nitish Behary Paray, Hiroyuki Sawatari, Syed Emir Irfan Wafa, Kamleshun Ramphul, Mushood Ahmed, Hritvik Jain, Saurabh Deshpande, Mohammed Khanji, Athol Umfrey Wells, Peter Collins, Selma Mohammed, Omar Abou-Ezzeddine, Vasilis Kouranos, Rakesh Sharma, Anwar Chahal

**Affiliations:** a Cardiac Sarcoidosis Services, Royal Brompton Hospital, Part of Guy’s and St Thomas’ NHS Trust, London, United Kingdom; b National Heart and Lung Institute, Imperial College London, London, United Kingdom; c Royal Devon University Healthcare NHS Foundation Trust, Exeter, United Kingdom; d Graduate School of Biomedical and Health Sciences, Hiroshima University, Hiroshima, Japan; e Russell’s Hall Hospital, Dudley Group NHS Foundation Trust, Dudley, United Kingdom; f Independent Researcher, Mauritius; g Rawalpindi Medical University, Rawalpindi, Pakistan; h Department of Internal Medicine, All India Institute of Medical Sciences (AIIMS), Jodhpur, India; i Department of Electrophysiology, Sri Jayadeva Institute of Cardiovascular Sciences and Research, Bangalore, India; j William Harvey Research Institute, NIHR Barts Biomedical Research Centre, Queen Mary University of London, Charterhouse Square, London EC1M 6BQ, United Kingdom; k Newham University Hospital, Barts Health NHS Trust, Glen Road, London E13 8SL, United Kingdom; l Barts Heart Centre, St Bartholomew’s Hospital, Barts Health NHS Trust, West Smithfield, London EC1A 7BE, United Kingdom; m Department of Cardiology, Creighton University, Omaha, USA; n Department of Cardiovascular Medicine, Mayo Clinic, 200 First Str, SW Rochester, MN 55905, USA; o Kings College London, London, United Kingdom; p Center for Inherited Cardiovascular Diseases, Department of Cardiology, WellSpan Health, 30 Monument Rd, York, PA 17403, USA

**Keywords:** Isolated cardiac sarcoidosis, Systemic sarcoidosis, National Readmissions Database

## Abstract

**Objective:**

To identify any differences in the characteristics and outcomes of patients with Isolated cardiac sarcoidosis (iCS) vs systemic cardiac sarcoidosis (sCS).

**Patients and methods:**

All inpatient encounters in the Nationwide Readmission Database from 2016 to 2021 were analyzed for the rates, predictors, costs and mortality during index and unplanned 90-days readmissions for iCS and sCS patients. Patients with ischemic heart disease were excluded.

**Results:**

1,667 patients were identified (57.8 % male), of which, 1,013 (60.8 %) had iCS and 654 (39.2 %) had sCS. The median (IQR) age of iCS patients was slightly older [57.0 (49.0–66.0) vs 56.0 (48.0–64.0), p = 0.04]. On index admission, iCS patients had higher prevalence of ventricular tachycardia (36.9 % vs 28.8 %, p = 0.001) and catheter ablation (5.6 % vs 2.8 %, p = 0.006). The predictors for all-cause readmissions were Charlson Comorbidity Index (CCI) (HR 1.19, 95 % CI 1.01–1.40, p = 0.04), age (HR 0.98 (0.97–1.00), p = 0.01) and the use of anticoagulant therapy (HR 1.92, 95 % CI 1.35–2.72, p < 0.001). Patients with sCS were more likely to be readmitted with heart failure compared to iCS patients (SHR 3.78, 95 % CI 1.11–12.94, p = 0.03). During subsequent readmission, iCS and sCS patients had comparable rates of in-hospital mortality, median length of stay and healthcare-associated costs. No independent predictors of in-hospital mortality at readmission were ascertained.

**Conclusions:**

Isolated CS patients, when compared to systemic CS, had a greater prevalence of ventricular tachycardia and catheter ablation. They were less likely to be re-hospitalized with heart failure within 90-days. Age, higher CCI, and use of anticoagulant therapy were predictors for all-cause readmissions.

## Graphical abstract

**Figure:**
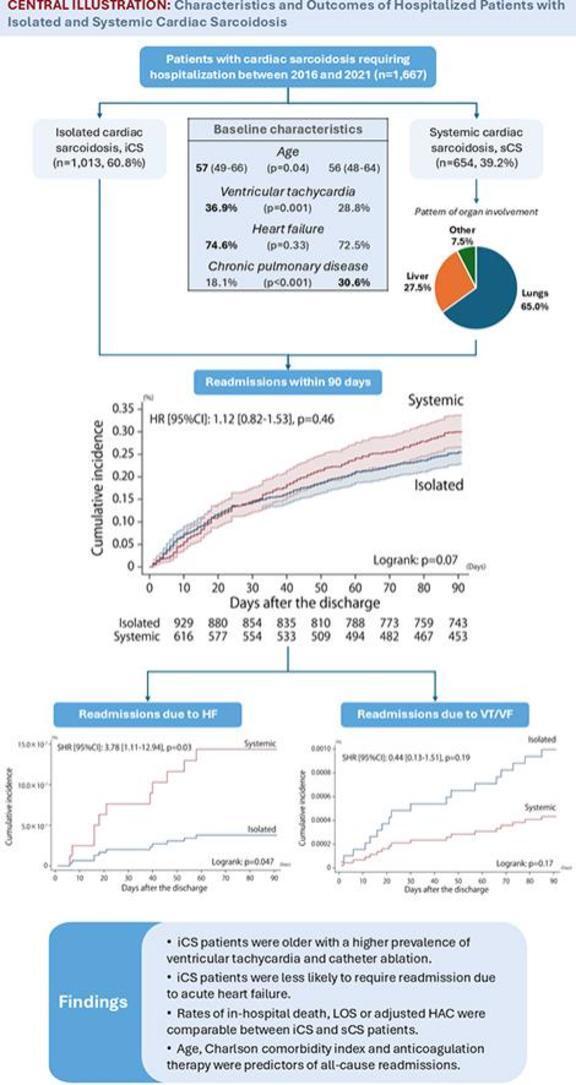

## 1 Introduction

Sarcoidosis is a multisystem inflammatory disease of unknown cause characterized by the formation of non-necrotizing granulomas in any organ but most commonly in the lungs, lymphatic system, skin, eyes, and heart[1], [2]. Despite being a relatively rare disease, sarcoidosis is marked by a high variability in incidence and prevalence between countries as well as within countries due to an intricate interplay of genetic, ethnic, racial, and environmental factors that is yet to be fully understood[3], [4], [5]. Clinically manifest cardiac involvement is estimated to affect approximately 1 in 20 patients with systemic sarcoidosis although the prevalence of cardiac sarcoidosis (CS) was found to be as high as 25 % in autopsy studies[6]. There is increasing recognition of the distinct clinical phenotype of isolated cardiac sarcoidosis (iCS), whereby cardiac manifestations happen without any apparent extracardiac involvement. Evidence is also emerging that patient with iCS are more likely to present with advanced heart disease and are at higher risk of adverse cardiac events, including mortality, compared to patients with concurrent extracardiac involvement or systemic CS (sCS)[7], [8], [9], [10].

### 1.1 Objectives

In this large-population real-world retrospective study using the Nationwide Readmission Database (NRD) from 2016 to 2021, the aim was to analyze the characteristics and outcomes of patients with iCS compared to patients with sCS on index admission and up to 90-days readmission. The hypothesis was that iCS patients were more likely to experience worse outcomes due to a higher incidence of adverse cardiac events. The objective was also to identify any predictors of readmission, length of hospital stay (LOS), healthcare-associated costs (HAC), and in-hospital mortality at readmission.

## 2 Methods

### 2.1 Database

Data was acquired from the NRD, which is part of the Healthcare Cost and Utilization Project (HCUP) and is monitored by the Agency for Healthcare Research and Quality (AHRQ). The NRD was established by federal state-industry partnership as an all-payer database with > 15 million discharge data points from 22 states. Unweighted data from the NRD accounts for approximately 51 % of the US population although subsequent adjustment based on city and hospital of origin allows for extrapolation for the national population[11]. Unique identification codes can link patients over 11 months, enabling readmissions to be captured. The anonymized nature of the data and retrospective nature of the study do not require Institutional Review Board approval.

### 2.2 Study population

The study population of interest was hospitalized patients aged 18 years or more with a diagnosis of CS admitted between 2016 and 2021 (eFig. 1). They were identified using the International Classification of Diseases, 10th Revision, Clinical Modification (ICD-10-CM) codes. Categorization of patients was based on the pattern of organ involvement of sarcoidosis (eTable 1). Where sarcoidosis involved only the heart, the case was categorized as iCS; where sarcoidosis involved the heart and at least 1 other organ, the case was categorized as sCS. Patients with the following characteristics were excluded: history or current ischemic heart disease, including its treatments such as angioplasty or coronary aortic bypass graft, missing data (i.e. age, sex, LOS, and HAC), combined transfer and/or two or more discharges on the same days, and hospitalizations in October, November, and December (due to unavailability of 90-day discharge follow-up data). Patients with the ICD 10 code of 86.9 (Sarcoidosis, unspecified) were also removed.

### 2.3 Variables

In addition to ICD-10-CM codes for discharge diagnoses, the NRD was queried for demographics, comorbidities, LOS, presence of in-hospital death, dates and reasons for initial admission, dates and reasons of readmission, hospital bed size, and primary payer. The data about types of comorbidities including types of cardiac implantable electronic devices, types of cardiac interventions, and presence of anti-coagulant therapy were abstracted based on ICD-10-CM/procedure code (eTable 2). Charlson comorbidity index (CCI) was calculated based on ICD-10-CM (eTable 3). The hospital bed size was categorized by three quantile range, which was categorized by HCUP and based on the region the hospital was located, area of the hospital, and teaching status of the hospital. Health insurance types were categorized as Medicare/Medicaid, private, and others. To estimate the cost of hospitalization more accurately, the NRD data were merged with Cost-to-Charge Ratios available from the HCUP. Adjusted healthcare-associated costs of each hospitalization were derived by multiplying the total hospital charge with Cost-to-Charge Ratios since the net medical costs included hospital bills for services such as wages, supplies, and utility that were different among the hospitals.

### 2.4 Statistical analysis

Continuous data were summarized as median with interquartile range (IQR) values; differences between the two groups were tested using the Mann–Whitney U tests after the Kolmogorov–Smirnov test. Categorical data were summarized as counts and percentages; differences between groups were tested using Pearson’s chi-squared tests. Multiple regression analysis was used for estimation of contributed factors for increasing of LOS and adjusted medical cost and described as standardized β. Logistic regression analysis was used to estimate the risk for in‐hospital mortality at readmission, shown as odds ratio (OR) and 95 % confidence interval (95 % CI). Regarding the survival analysis, Kaplan-Meier analysis and log-rank test on non-weighted NRD results were used to compare the rate and timing of 90-day readmission between iCS and sCS groups. Cox proportional hazard regression analysis was used for risk factors for readmission. For competing risks analysis, Fine-Gray sub distribution hazard model was used. The results of these multivariate survival analysis were shown as hazard ratio (HR) or sub-hazard ratio (SHR) and 95 % CI. The included variables for the multivariate analysis (i.e. multiple regression analysis, logistic regression analysis, cox proportional hazard regression analysis, and Fine-Gray sub distribution hazard model) were age, sex, CCI, presence of anti-coagulant therapy and admission due to cardiac arrest, types of sarcoidosis (i.e., iCS or sCS), cardiac interventions, and comorbidities, hospital bed size, and health insurance types. As per NRD rules for protecting personal information, to prevent identification, some cases were also blinded if n ≤ 10 in a particular hospital or area were present. Analysis was done using STATA v.15.1 (Stata‐Corp). A two‐tailed priori p < 0.05 was regarded as significant.

## 3 Results

### 3.1 Index admission

Over the 6-year period between 2016 and 2021, 1,667 CS admissions were identified using the inclusion criteria (eFig. 1). Of these, 1013 (60.8 %) had iCS. The pattern of organ involvement other than the heart in patients with CS is summarized in eTable 4. In patients with sCS (n = 654), the lungs were the most affected organ (65.0 %) followed by the liver (27.5 %), a combination of the lungs and lymph nodes (4.0 %), and the skin (2.3 %).

The baseline characteristics and comorbidities for the entire cohort are provided in Table 1. At first hospitalization, the median age (IQR) of patients was 57.0 (49.0–65.0) years with iCS patients being slightly older: iCS 57.0 (49.0–66.0) years vs sCS 56.0 (48.0–64.0) years, p = 0.04. There was a preponderance of males in both groups, iCS 58.1 % vs sCS 57.3 %, p = 0.78. The iCS group demonstrated lower median Charlson Comorbidity index (CCI) 2.0 (1.0–4.0) compared to the sCS group 3.0 (2.0–4.0), although this difference only trended to statistical significance (p = 0.08). History of ventricular tachycardia (36.9 % vs 28.8 %, p = 0.001) and catheter ablation (5.6 % vs 2.8 %, p = 0.006) were more prevalent in the iCS group. Conversely, chronic pulmonary disease (18.1 % vs 30.6 %, p < 0.001) and obesity (24.7 % vs 30.4 %, p = 0.01) were more prevalent in the sCS group. No significant differences were detected between the two groups regarding the prevalence of heart failure, use of anticoagulant therapy, atrioventricular block, atrial fibrillation, presence of cardiac implantable devices, cerebrovascular diseases, peripheral vascular diseases, liver diseases, diabetes, renal diseases, hypertension, coagulation defects and history of cardiac transplant (Table 1). 50.9 % of patients paid using Medicare/Medicaid while 44.8 % were funded by private insurance (p = 0.008). iCS patients were less likely to use Medicare/Medicaid (48.1 % vs 55.2 %) and have recourse to private insurance (47.8 % vs 40.1 %) compared to sCS patients. Patients with iCS had a similar median length of stay compared to sCS patients: 4 days (2–7) vs 4 days (2–8), p = 0.001.

**Table 1:** Baseline characteristics at first admission.

	All	Systemic	Isolated	P-value
Number, N (%)	1,667 (100.0)	654 (39.2)	1,013 (60.8)	−
Age, years	57.0 (49.0–65.0)	56.0 (48.0–64.0)	57.0 (49.0–66.0)	0.04
Male, N (%)	963 (57.8)	375 (57.3)	588 (58.1)	0.78
Charlson comorbidity index, points	3.0 (1.0–4.0)	3.0 (2.0–4.0)	2.0 (1.0–4.0)	0.08
Anti-coagulant therapy, N (%)	464 (27.8)	181 (27.7)	283 (27.9)	0.91
Length of stay, day	4.0 (2.0–7.0)	4.0 (2.0–8.0)	4.0 (2.0–7.0)	0.001
Cardiac arrest, N (%)	25 (1.5)	<10 (N/A)	<20 (N/A)	0.46
In-hospital death, N (%)	26 (1.6)	<10 (N/A)	<20 (N/A)	0.20
**HAC, US$**				
Net value	55,261.0(22,906.0–127,271.0)	54,664.0(25,885.0–122,960.0)	56,138.0(21,245.0–131,403.0)	0.94
Adjusted value	14,820.6(7,212.7–34,013.9)	15,059.8(7,516.6–31,468.0)	14,519.1(7,014.0–36,125.9)	0.61
**CIED implantation, N (%)**				
PPM	36 (2.2)	13 (2.0)	23 (2.3)	0.70
ICD	168 (10.1)	65 (9.9)	103 (10.2)	0.88
CRT-P	<10 (N/A)	<10 (N/A)	<10 (N/A)	0.26
CRT-D	85 (5.1)	28 (4.3)	57 (5.6)	0.22
**Cardiac interventions, N (%)**				
Catheter ablation	75 (4.5)	18 (2.8)	57 (5.6)	0.006
Cardiac transplant	49 (2.9)	15 (2.3)	34 (3.4)	0.21
**Comorbidities, N (%)**				
Ventricular tachycardia	562 (33.7)	188 (28.8)	374 (36.9)	0.001
Ventricular fibrillation	<10 (N/A)	<10 (N/A)	<10 (N/A)	0.41
Atrioventricular block	341 (20.5)	123 (18.8)	218 (21.5)	0.18
Sick sinus syndrome	37 (2.2)	<10 (N/A)	<30 (N/A)	0.03
Atrial fibrillation	469 (28.1)	178 (27.2)	291 (28.7)	0.50
Heart failure	1,230 (73.8)	474 (72.5)	756 (74.6)	0.33
Cerebrovascular diseases	52 (3.1)	17 (2.6)	35 (3.5)	0.33
Chronic Pulmonary disease	383 (23.0)	200 (30.6)	183 (18.1)	<0.001
Peripheral vascular diseases	750 (45.0)	275 (42.1)	475 (46.9)	0.052
Liver diseases	72 (4.3)	33 (5.1)	39 (3.9)	0.24
Diabetes	433 (26.0)	185 (28.3)	248 (24.5)	0.08
Renal diseases	450 (27.0)	175 (26.8)	275 (27.2)	0.86
Hypertension	445 (26.7)	187 (28.6)	258 (25.5)	0.16
Coagulation defects	33 (2.0)	12 (1.8)	21 (2.1)	0.73
Obesity	449 (26.9)	199 (30.4)	250 (24.7)	0.01
**Hospital bed size, N (%)**				
Small	76 (11.0)	29 (11.7)	47 (10.6)	
Medium	118 (17.1)	34 (13.7)	84 (19.0)	
Large	497 (71.9)	186 (74.7)	311 (70.4)	0.20
**Primary payer, N (%)**				
Medicare/Medicaid	848 (50.9)	361 (55.2)	487 (48.1)	
Private insurance	746 (44.8)	262 (40.1)	484 (47.8)	
Others	72 (4.3)	31 (4.7)	41 (4.1)	0.008

**HAC**: healthcare-associated costs; **CIED**: cardiac implantable electronic devices; **PPM**: permanent pacemaker; **CRT-P**: cardiac resynchronization therapy with pacemaker; **CRT-D**: cardiac resynchronization therapy with defibrillator.

Multivariable analysis did not yield any independent clinical predictors of in-hospital mortality on the first hospitalization (Table 2). At the index admission, CCI (hazards ratio [HR] 1.19 (1.01–1.40), p = 0.04), age (HR 0.98 (0.97–1.00), p = 0.01) and anticoagulant therapy (HR 1.92, 95 % CI 1.35–2.72, p < 0.001) were predictors of readmission (Table 3).

**Table 2:** Predictors of increasing LOS, HAC, and in-hospital death at 1st admission.

	LOS		HAC (Adjusted)		In-hospital death	
	β	P-value	β	P-value	OR (95 % CI)	P-value
Systemic sarcoidosis	0.03	0.32	0.004	0.87	0.61 (0.08–4.59)	0.63
Age	0.02	0.68	−0.005	0.86	1.07 (0.98–1.17)	0.12
Male	0.02	0.48	−0.006	0.82	2.33 (0.40–13.75)	0.35
Charlson comorbidity index	0.03	0.76	0.001	0.99	−	−
Anti-coagulant therapy	−0.001	0.97	0.02	0.47	0.34 (0.04–2.61)	0.30
Cardiac arrest	−	−	−	−	−	−
**CIED implantation**						
PPM	−0.001	0.98	0.05	0.06	−	−
ICD	0.04	0.28	0.17	<0.001	−	−
CRT-P	−	−	−	−	−	−
CRT-D	0.03	0.43	0.16	<0.001	−	−
**Cardiac interventions**						
Catheter ablation	0.001	0.97	0.06	0.02	−	−
Cardiac transplant	0.47	<0.001	0.70	<0.001	8.18 (0.43–155.90)	0.16
**Comorbidities**						
Ventricular tachycardia	0.04	0.28	0.04	0.11	1.56 (0.20–11.81)	0.67
Ventricular fibrillation	−	−	−	−	−	−
Atrioventricular block	−0.003	0.94	0.01	0.61	1.38 (0.10–19.39)	0.81
Sick sinus syndrome	−0.003	0.94	−0.02	0.52	−	−
Atrial fibrillation	−0.01	0.78	0.01	0.72	2.84 (0.42–19.22)	0.28
Heart failure	0.09	0.03	0.03	0.46	−	−
Cerebrovascular diseases	0.004	0.92	0.07	0.02	−	−
Chronic Pulmonary disease	0.0004	0.99	−0.01	0.65	−	−
Peripheral vascular diseases	−0.12	0.005	−0.04	0.17	−	−
Liver diseases	0.07	0.04	0.03	0.23	−	−
Diabetes	−0.02	0.77	−0.01	0.81	−	−
Renal diseases	0.12	0.02	0.10	0.02	−	−
Hypertension	−0.01	0.72	−0.02	0.59	−	−
Coagulation defects	0.03	0.40	−0.02	0.36	−	−
Obesity	−0.02	0.58	−0.03	0.33	1.02 (0.14–7.65)	0.99
**Hospital bed size**						
Small	(ref.)	−	(ref.)	−	(ref.)	−
Medium	0.09	0.051	0.009	0.81	0.51 (0.07–7.65)	0.50
Large	0.10	0.04	0.05	0.18	0.041 (0.003–0.481)	0.01
**Primary payer**						
Medicare/Medicaid	(ref.)	−	(ref.)	−	(ref.)	−
Private insurance	−0.03	0.47	0.003	0.92	0.47 (0.04–5.43)	0.55
Others	0.04	0.23	0.02	0.48	−	−

**LOS**: length of stay; **HAC**: healthcare-associated costs; **OR**: odds ratio; **CI**: confidence interval; **CIED**: cardiac implantable electronic devices; **PPM**: permanent pacemaker; **CRT-P**: cardiac resynchronization therapy with pacemaker; **CRT-D**: cardiac resynchronization therapy with defibrillator.

**Table 3:** Predictors of readmission at first admission.

	HR (95 % CI)	P-value
Systemic sarcoidosis	1.13 (0.83–1.55)	0.43
Age	0.98 (0.97–1.00)	0.01
Male	1.01 (0.73–1.39)	0.95
Charlson comorbidity index	1.19 (1.01–1.40)	0.04
Anti-coagulant therapy	1.92 (1.35–2.72)	<0.001
Cardiac arrest	−	−
**CIED implantation**		
PPM	0.83 (0.19–3.61)	0.81
ICD	1.06 (0.59–1.91)	0.85
CRT-P	−	−
CRT-D	1.05 (0.53–2.07)	0.90
**Cardiac interventions**		
Catheter ablation	1.14 (0.49–2.67)	0.76
Cardiac transplant	1.68 (0.81–3.51)	0.16
**Comorbidities**		
Ventricular tachycardia	0.94 (0.66–1.34)	0.74
Ventricular fibrillation	−	−
Atrioventricular block	0.99 (0.67–1.48)	0.98
Sick sinus syndrome	0.73 (0.27–2.01)	0.55
Atrial fibrillation	0.70 (0.48–1.03)	0.07
Heart failure	1.10 (0.69–1.76)	0.69
Cerebrovascular diseases	1.42 (0.70–2.85)	0.33
Chronic Pulmonary disease	0.77 (0.51–1.16)	0.21
Peripheral vascular diseases	0.81 (0.56–1.18)	0.28
Liver diseases	0.49 (0.21–1.16)	0.11
Diabetes	0.89 (0.49–1.61)	0.69
Renal diseases	1.04 (0.66–1.64)	0.86
Hypertension	1.20 (0.80–1.78)	0.38
Coagulation defects	1.34 (0.47–3.79)	0.59
Obesity	0.95 (0.67–1.35)	0.78
**Hospital bed size**		
Small	(ref.)	−
Medium	1.35 (0.71–2.57)	0.36
Large	1.37 (0.79–2.38)	0.27
**Primary payer**		
Medicare/Medicaid	(ref.)	−
Private insurance	0.73 (0.53–1.02)	0.07
Others	0.79 (0.34–1.85)	0.59

**HR:** hazards ratio; **CI**: confidence interval; **CIED**: cardiac implantable electronic devices; **PPM**: permanent pacemaker; **CRT-P**: cardiac resynchronization therapy with pacemaker; **CRT-D**: cardiac resynchronization therapy with defibrillator.

### 3.2 Up-to-90-day readmission

447 patients required readmission within 90 days, of whom 194 had sCS (30.0 % of the sCS cohort) and 253 had iCS (25.5 % of the iCS cohort) (sCS: HR 1.13 (0.83–1.55), p = 0.43). When the cohorts of iCS and sCS patients are considered separately, those who required hospitalization within 90-days in each group demonstrated higher CCI, use of anti-coagulant therapy, co-existence of heart failure, diabetes, and renal diseases (eTable 5). Readmission was associated with longer LOS in both groups, although no significant differences were found in adjusted HAC. In the iCS group, history of cardiac transplant (5.9 % vs 2.4 %, p = 0.007) was seen more frequently in those needing readmission. In the sCS group, presence of an implantable cardioverter-defibrillator (ICD) was seen more commonly in patients not requiring readmission (11.7 % vs 6.2 %, p = 0.03) while atrial fibrillation (24.3 % vs 34.5 %, p = 0.007) and peripheral vascular diseases (39.7 % vs 49.0 %, p = 0.03) were more prevalent in those needing readmission.

**Fig. 1:**
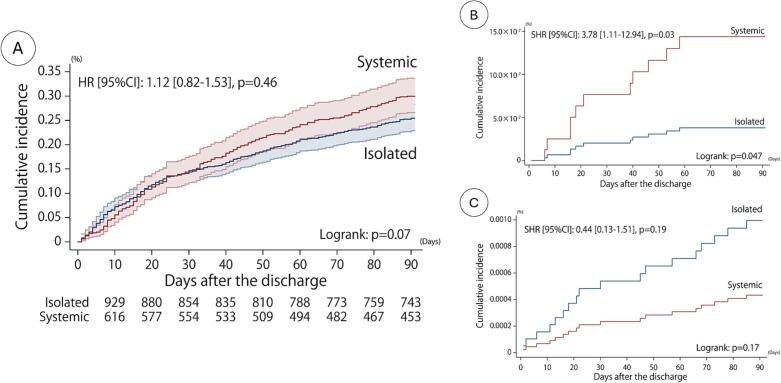
Kaplan-Meier curve of cumulative incidence of readmission with 90 days. Curve A demonstrates all-cause readmissions. Curve B demonstrates readmissions due to heart failure. Curve C demonstrates readmissions due to VT/VF.

The Kaplan-Meier curve in Fig. 1 demonstrates numerically higher incidence of all-cause readmission among sCS patients compared to iCS patients although the difference between the two groups only trended towards statistical significance (HR 1.12, 95 % CI 0.82–1.53, logrank p = 0.07). Sub-group analyses based on cause for readmission demonstrated that, compared to iCS patients, sCS patients had a higher incidence of hospitalizations within 90 days due to heart failure (SHR 3.78, 95 % CI 1.11–12.94, p = 0.03). No significant difference was found in the incidence of 90-day readmission due to ventricular tachycardia or fibrillation (VT/VF) between the two groups (SHR 0.44, 95 % CI 0.13–1.51, p = 0.19).

When compared to each other, patients with iCS vs sCS did not differ in the incidence of in-hospital death (1.3 % vs 1.6 %, p = 0.69), median LOS (4[2], [3], [4], [5], [6] vs 4 [2], [3], [4], [5], [6], [7], p = 0.17) and adjusted HAC ($13,702.9[6,766.3–32,780.2] vs $12,919.3[6,689.9–28,633.2], p = 0.41) (Table 4).

**Table 4:** Differences between patients with isolated sarcoidosis and patients with systemic sarcoidosis at readmission.

	Systemic	Isolated	P-value
In-hospital death, N (%)	10 (1.6)	13 (1.3)	0.69
Length of stay, day	4.0 (2.0–7.0)	4.0 (2.0–6.0)	0.17
**HAC, US$**			
Net value	47,175.0(22,376.0–110,164.0)	48,742.0(20,735.0–120,675.0)	0.43
Adjusted value	12,919.3(6,689.9–28,633.2)	13,702.9(6,766.3–32,780.2)	0.41

**HAC**: healthcare-associated cost.

At readmission, on multivariable analysis, a history of cardiac transplant was identified as a predictor of increasing LOS and higher HAC (eTable 6). No independent predictors of in-hospital death were found.

## 4 Discussion

In the present study of 1,667 hospitalized patients with cardiac sarcoidosis, the implications of iCS on clinical outcomes and readmissions using a nationally representative database were analyzed. To our knowledge, this is the largest real-world study to do so. The main findings of the study were as follows:

I. A sizeable majority of the cohort of CS patients who required hospitalization between 2016 and 2021 had iCS (60.8 %).
II. Compared to sCS patients, patients with iCS were slightly older and more likely to have a history of VT or catheter ablation.
III. Among the variables analyzed in this study, no independent predictors of in-hospital mortality among CS patients were identified.
IV. sCS patients were more likely than iCS patients to require readmission within 90 days due to heart failure.
V. Age, CCI and use of anticoagulation therapy were associated with higher incidence of all-cause readmissions.

Historically, the prevalence of iCS varied significantly between 3 % to 65 %, reflecting the differences in definitions and clinical criteria used for diagnosis[8], [12], [13]. With the advancement of technology and refinement of protocols, cardiac magnetic resonance imaging (CMR) and cardiac fluoro-deoxy-glucose positron emission tomography (FDG-PET) have emerged as powerful tools in the diagnostic evaluation of patients with suspected cardiac sarcoidosis (isolated or systemic)[14], [15], [16], [17]. This led to their inclusion among major diagnostic criteria in the latest Heart Rhythm Society (HRS), Japanese Circulation Society (JCS) and American Heart Association (AHA) guidelines[18], [19], [20]. The increasing availability of these resources has driven a paradigm shift such that CS, which has a notoriously varied and non-specific clinical presentation, is now detected at an earlier stage where there is no extracardiac involvement[21]. Once CS is diagnosed, patients are likely to start treatment which may further delay manifestation in other organs. This study analysis seems to corroborate this hypothesis as 60.8 % of inpatient CS patients were found to have iCS. However, this observation has to be considered with 2 caveats and may not necessarily be reflective of the actual prevalence of iCS among all CS patients in the community. Firstly, patients with history of ischemic heart disease and whose conditions were not appropriately coded in the NRD, for example patients with “unspecified sarcoidosis” code, were excluded from the statistical analysis in order to improve the accuracy and reliability of the data pool. Secondly, the study focuses on inpatients only, which the authors postulate is due to iCS patients requiring admission more frequently than sCS patients.

The observation that iCS patients were noted to be older than sCS patients could possibly stem from the diagnostic delay that iCS patients experience compared to those in whom sarcoidosis was associated with other organ involvement. In their single-center retrospective study, Sink *et al.* identified that iCS patients were significantly more likely to experience a delay of > 6 months in the diagnosis of CS from the first documented episode consistent with highly probable or probable cardiac involvement, especially in cases where the presentation mimicked that of idiopathic non-ischemic dilated cardiomyopathy[9]. Another observation that could be attributed to the diagnostic delay that iCS patients experience is the higher prevalence of VT and history of catheter ablation in this cohort[22]. This may reflect the established tendency for clinicians to assess for more common causes of ventricular arrhythmias, such as ischemic cardiomyopathy, electrolyte abnormalities, and inherited channelopathies, in the first instance and delay investigations for cardiac sarcoidosis after the former have been excluded. A delayed diagnosis of CS is associated with higher risks of adverse events and poorer outcomes[23], [24]. Interestingly, at index admission, advancing age was associated with a lower incidence of readmission, which likely reflects the lower readmission rates among the older cohort of iCS patients rather than advancing age being a protective factor *per se*. Similarly, the authors hypothesize that higher CCI and use of anticoagulation therapy predicting all-cause readmissions in the two cohorts together likely demonstrate those CS patients who were more comorbid and did not necessarily get admitted for cardiac causes only.

The results of the present study do not demonstrate a significant difference in the history of heart failure, atrial fibrillation or use of implantable defibrillators between iCS and sCS patients, which is not consistent with previous findings. The discrepancy in the observed prevalence of certain cardiovascular comorbidities could be partially attributed to the difference in the ethnicity of the population studied. While the present study involved a representative sample of the US population, studies from Sato, Okada, Takaya and colleagues were based on Japanese participants only[25], [26], [27]. Rather than a single disease entity, it is probable that CS may be a constellation of closely-related conditions, each with a characteristic phenotype, influenced by the triggering exposure and underlying genotype[28], [29]. Tan *et al.* identified the association of female sex, Black ancestry and socioeconomic deprivation with higher age-adjusted mortality rates in their analysis of non-ischemic cardiovascular mortality in patients with sarcoidosis from 1999 to 2020[30], [31]. The current study was not specifically designed to examine the influence of demographic and social factors in the manifestations and outcomes of CS but it is possible that these could underlie some of the observations.

CS can manifest as heart failure with preserved or reduced ejection fraction, especially if myocardial involvement is extensive. Heart failure is considered to be the most important cause of death from CS, accounting for up to 25 % of CS-related mortality in some studies[10], [32]. In the Finnish registry studies by Kandolin *et al*. involving patients with histologically-proven CS, 9 %-27 % of cases reported clinical signs and symptoms of heart failure while 59 %-82 % were diagnosed with left ventricular systolic dysfunction[7], [32]. In the present study, it was established that sCS patients were more likely to require readmission to hospital within 90 days due to heart failure compared to iCS patients. By contrast, In a recent secondary analysis of the multicenter registry of Japanese patients with CS, Maeda *et al*. observed that the risk of all-cause death, hospitalization for heart failure, or fatal ventricular arrhythmia events were comparable between iCS and sCS after prognostic covariates were adjusted for[33]. Of note, sCS patients in the present study demonstrated higher prevalence of chronic pulmonary disease and obesity. This reflects the pattern of extra-cardiac involvement observed in sCS patients in whom the lungs are the most affected organ in addition to the heart. It is possible that, in sCS patients, right ventricular (RV) involvement of CS is more common, not only as a consequence of direct granulomatous disease of the RV myocardium but also due to the hemodynamic stress of longstanding pulmonary hypertension in pulmonary sarcoidosis[34], [35].

### 4.1 Strengths and limitations

The large number of patients across multiple centers in the US analyzed is one of the major strengths of this study. Furthermore, by excluding patients with ischemic heart disease, the study limits the effect of this significant confounding factor on the cardiovascular outcomes studied[36]. By excluding patients with the ICD-10-CM code of 86.9 (Sarcoidosis, unspecified), the study follows a rigorous methodology of only including those cases in which organ involvement of sarcoidosis is known.

However, the findings need to be considered within a few limitations. The objective was to study a cohort of CS patients who required hospitalization. The findings may not necessarily apply to patients with stable CS who are predominantly managed in outpatient settings. The findings cannot be extrapolated beyond 90-day follow-up. There was no access to histological or advanced imaging records to confirm the diagnosis of CS. Isolated CS does not have a specific ICD-10-CM code. Patients with iCS were identified as those having ICD-10-CM codes for cardiac sarcoidosis and no codes for sarcoidosis affecting other organs. By contrast, sCS patients had codes for both cardiac sarcoidosis and sarcoidosis in at least one other organ. It is impossible to fully resolve any errors in clinical coding which are intrinsic to large databases. For example, some of the patients included in the iCS cohort may have had inflammatory cardiomyopathy presenting with VT and requiring ablation[37]. VT itself may cause regional inflammation, mimicking an uptake pattern on FDG-PET traditionally associated with iCS[38]. Similarly, regional myocarditis, especially during the Covid-19 pandemic, could have contributed to the high prevalence of iCS and observed worse outcomes. Due to the lack of information about heart failure and immunosuppressive therapies available to patients, it is not possible to assess the influence of differences in treatment strategy on patient outcomes[39], [40], [41], [42], [43]. Moreover, NRD dataset does not provide data on ethnicities of patients, which is quite relevant in a heterogeneous condition like cardiac sarcoidosis. Lastly, it is plausible that iCS is an earlier stage of systemic sarcoidosis which implies that, over the study period, some iCS patients may have developed sCS. Due to the lack of long-term follow-up data, this study does not have the data granularity to identify these patients and analyze them separately. Further research could benefit from longer follow-up periods and more detailed data on treatment regimens and patient demographics.

## 5 Conclusion

In patients with CS hospitalized in the US, mortality, length of stay and healthcare costs were comparable between the two cohorts of iCS and sCS with no significant predictor for in-hospital mortality. Patients with iCS were slightly older, had higher prevalence of ventricular tachycardia and catheter ablation, but a lower prevalence of chronic pulmonary disease and obesity with a lower incidence of acute heart failure admissions. Overall age, a higher Charlson Comorbidity Index and use of anticoagulation therapy were associated with all-cause readmissions.

## CRediT authorship contribution statement

**Raheel Ahmed:** Writing – review & editing, Writing – original draft, Visualization, Resources, Project administration, Investigation, Formal analysis, Data curation, Conceptualization. **Nitish Behary Paray:** Writing – review & editing, Writing – original draft, Supervision, Methodology, Investigation, Formal analysis, Conceptualization. **Hiroyuki Sawatari:** Writing – review & editing, Validation, Software, Methodology, Formal analysis, Data curation, Conceptualization. **Syed Emir Irfan Wafa:** Writing – review & editing, Validation, Resources, Data curation. **Kamleshun Ramphul:** Validation, Software, Methodology, Formal analysis. **Mushood Ahmed:** Validation, Software, Data curation, Conceptualization. **Hritvik Jain:** Writing – review & editing, Validation, Project administration, Methodology. **Saurabh Deshpande:** Writing – review & editing, Visualization, Project administration, Methodology, Conceptualization. **Mohammed Khanji:** Writing – review & editing, Validation, Investigation, Data curation, Conceptualization. **Athol Umfrey Wells:** Writing – review & editing, Validation, Methodology, Conceptualization. **Peter Collins:** Writing – review & editing, Methodology, Formal analysis, Conceptualization. **Selma Mohammed:** Writing – review & editing, Investigation, Data curation, Conceptualization. **Omar Abou-Ezzeddine:** Writing – review & editing, Validation, Investigation, Data curation. **Vasilis Kouranos:** Writing – review & editing, Validation, Resources, Methodology, Conceptualization. **Rakesh Sharma:** Writing – review & editing, Writing – original draft, Validation, Supervision, Methodology, Formal analysis, Conceptualization. **Anwar Chahal:** Writing – review & editing, Writing – original draft, Validation, Supervision, Project administration, Methodology, Investigation, Data curation, Conceptualization.

## Declaration of competing interest

The authors declare that they have no known competing financial interests or personal relationships that could have appeared to influence the work reported in this paper.
