# Supplementary material for: Characteristics and outcomes of hospitalized patients with Isolated and systemic cardiac sarcoidosis: Analysis of the Nationwide readmissions database 2016–2021
Source: Int J Cardiol Heart Vasc. 2025 Feb 24;57:101636. doi: 10.1016/j.ijcha.2025.101636 (PMC11907453; doi:10.1016/j.ijcha.2025.101636)
Supplement: Supplementary Data 1 [file mmc1.docx]

**SUPPLEMENTAL FIGURE AND TABLES**

**eFigure 1. Patient selection flowchart.**


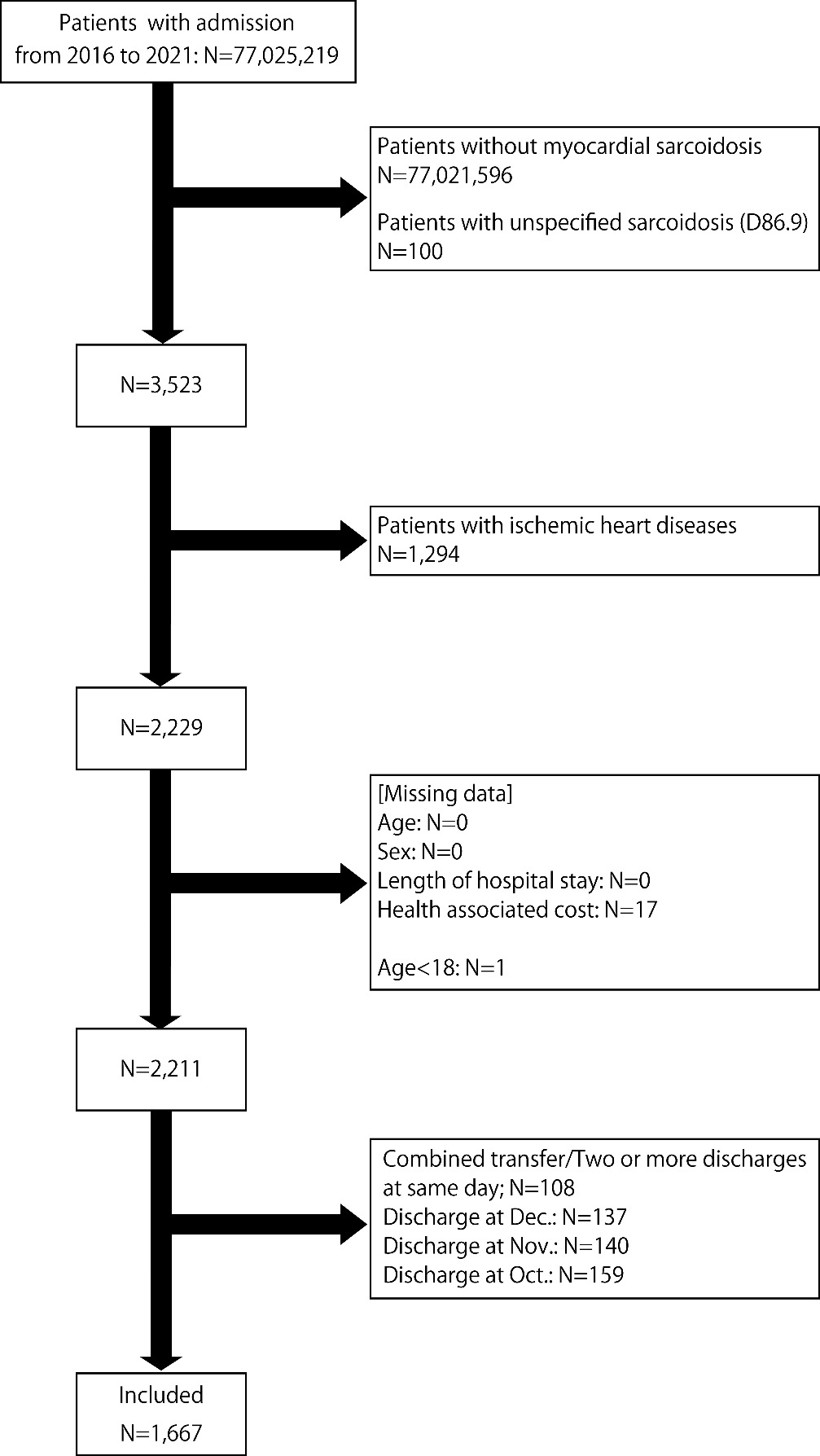


**eTable 1 ICD-10-CM codes for isolated and systemic cardiac sarcoidosis**

|  | **Isolated** | **Systemic** |
| --- | --- | --- |
| Sarcoidosis | D86.85 only | D86.85+ at least one from:  D86.0, D86.1, D86.2, D86.3, D86.81, D86.82, D 86.83, D86.84, D86.86, D86.87, D86.89 |

ICD-10-CM: The International Classification of Diseases, Tenth Revision, Clinical Modification

**eTable 2 ICD-10-CM and procedure codes for comorbidities and treatments**

| Cardiac arrest | 5A12012*, I46.2, I46.8, I46.9 |
| --- | --- |
| Anti-coagulant therapy | XW03392*, XW04392*, 3E033PZ*, 3E043PZ*, 3E053PZ*, 3E063PZ*, 3E083PZ*, Z7901*, Z7902* |
| **CIED implantation** |  |
| PPM | 0JH605Z*, 0JH805Z*, 0JH835Z*, 0JH606Z*, 0JH636Z*, 0JH806Z*, 0JH836Z*, 0JH604Z*, 0JH634Z*, 0JH804Z*, 0JH834Z*, 02HL3JZ*, 02HL4JZ* |
| ICD | 0JH608Z*, 0JH638Z*, 0JH808Z*, 0JH838Z* |
| CRT-P | 0JH607Z*, 0JH637Z*, 0JH807Z*, 0JH837Z* |
| CRT-D | 0JH609Z*, 0JH639Z*, 0JH809Z*, 0JH839Z* |
| **Cardiac interventions** |  |
| Catheter ablation | 02583ZZ*, 02B83ZZ*, 02T83ZZ* |
| Cardiac transplant | 02YAx*, 02RKx*, 02RLx* |
| **Comorbidities** |  |
| Ventricular tachycardia | I47.2 |
| Ventricular fibrillation | I49.1 |
| Atrioventricular block | I44.0, I44.1, I44.2. I44.30, I44.39 |
| Sick sinus syndrome | I4.95 |
| Atrial fibrillation | I48.0, I48.1, I48.2, I48.91 |
| Heart failure | I11.0, I13.0, I13.2, I50.1, I50.2x, I50.3x, I50.4x, I50.81x, I50.82, I50.83, I50.84, I50.89, I50.9 |
| Cerebrovascular diseases | G45.x, G46.x, H34.0x, H34.1x, H34.2x, I60.x, I61.x, I62.x, I63.x, I64.x, I65.x, I66.x, I67.x, I68.x |
| Chronic Pulmonary disease | J40.x, J41.x, J42.x, J43.x, J44.x, J45.x, J46.x, J47.x, J60.x, J61.x, J62.x, J63.x, J64.x, J65.x, J66.x, J67.x, J68.4, J70.1, J70.3 |
| Peripheral vascular diseases | I70.x, I71.x, I73.1, I73.8x, I73.9, I77.1, I79.0, I79.1, I79.8, K55.1, K55.8, K55.9, Z95.8x, Z95.9 |
| Liver diseases | B18.x, K70.0, K70.1x, K70.2, K70.3x, K70.9, K71.3, K71.4, K71.5x, K71.7, K73.x, K74.x, K76.0, K76.2, K76.3, K76.4, K76.8x, K76.9, Z94.4, I85.0x, I86.4, K70.4x, K71.1x, K72.1x, K72.9x, K76.5, K76.6, K76.7 |
| Diabetes | E08.0x, E08.1x, E08.6x, E08.8, E08.9, E09.0x, E09.1x, E09.6x, E09.8x, E09.9x, E10.1x, E10.6x, E10.8, E10.9, E11.0x, E11.1x, E11.6x, E11.8, E11.9, E13.0x, E13.1x, E13.6x, E13.8, E13.9, E08.2x, E08.3x, E08.4x, E08.5x, E09.2x, E09.3x, E09.4x, E09.5x, E10.2x, E10.3x, E10.4x, E10.5x, E11.2x, E11.3x, E11.4x, E11.5x, E13.2x, E13.3x, E13.4x, E13.5x, |
| Renal diseases | I12.0, I12.9, I13.0, I13.10, I13.11, I13.2, N03.x, N05.x, N18.1, N18.2, N18.3x, N18.4, N18.5, N18.6, N18.9, N19, N25.0, Z49.x, Z99.2 |
| Hypertension | H35.03x, I10, I11.9, I12.0, I12.9, I13.10, I13.11, I15.x, I16.x, I67.4, O10.01x, O10.02, O10.03, O10.11x, O10.12, O10.13, O10.21x, O10.22, O10.23, O10.31x, O10.32, O10.33, O10.41x, O10.42, O10.43, O10.91x, O10.92, O10.93, O11.x, O16.x |
| Coagulation defects | D66, D68.0, D68.1, D68.2, D68.318, D68.59, D68.8, D68.9 |
| Obesity | E66.9, Z68.3x, Z68.41, Z68.42, Z68.43, Z68.44, Z68.45 |

ICD-10-CM: The International Classification of Diseases, Tenth Revision, Clinical Modification, CIED: cardiovascular implantable electronic device, PPM: permanent pacemaker, ICD: implantable cardioverter-defibrillator, CRT-P: cardiac resynchronization therapy with pacemaker, CRT-D: cardiac resynchronization therapy with defibrillator

*Abstracted as procedure codes.

**eTable 3 ICD-10-CM codes for Charlson comorbidity index**

| **Diagnosis** | **ICD-10-CM code** |
| --- | --- |
| Congestive heart failure (Myocardial infarction) | I21.x, I22.x. I25.2 |
| Congestive heart failure (Excluding Myocardial infraction) | I11.0, I13.0, I13.2, I25.5, I42.0, I42.5, I42.6, I42.7, I42.8, I42.9, I43.x, I50.x, P29.0 |
| Cerebrovascular diseases | G45.x, G46.x, H34.0x, H34.1x, H34.2x, I60.x, I61.x, I62.x, I63.x, I64.x, I65.x, I66.x, I67.x, I68.x |
| Chronic Pulmonary disease | J40.x, J41.x, J42.x, J43.x, J44.x, J45.x, J46.x, J47.x, J60.x, J61.x, J62.x, J63.x, J64.x, J65.x, J66.x, J67.x, J68.4, J70.1, J70.3 |
| Peripheral vascular diseases | I70.x, I71.x, I73.1, I73.8x, I73.9, I77.1, I79.0, I79.1, I79.8, K55.1, K55.8, K55.9, Z95.8x, Z95.9 |
| Liver disease (Mild) | B18.x, K70.0, K70.1x, K70.2, K70.3x, K70.9, K71.3, K71.4, K71.5x, K71.7, K73.x, K74.x, K76.0, K76.2, K76.3, K76.4, K76.8x, K76.9, Z94.4 |
| Liver disease (Moderate or severe) | I85.0x, I86.4, K70.4x, K71.1x, K72.1x, K72.9x, K76.5, K76.6, K76.7 |
| Diabetes (without any complications) | E08.0x, E08.1x, E08.6x, E08.8, E08.9, E09.0x, E09.1x, E09.6x, E09.8x, E09.9x, E10.1x, E10.6x, E10.8, E10.9, E11.0x, E11.1x, E11.6x, E11.8, E11.9, E13.0x, E13.1x, E13.6x, E13.8, E13.9 |
| Diabetes (with chronic complications) | E08.2x, E08.3x, E08.4x, E08.5x, E09.2x, E09.3x, E09.4x, E09.5x, E10.2x, E10.3x, E10.4x, E10.5x, E11.2x, E11.3x, E11.4x, E11.5x, E13.2x, E13.3x, E13.4x, E13.5x, |
| Renal diseases | I12.0, I12.9, I13.0, I13.10, I13.11, I13.2, N03.x, N05.x, N18.1, N18.2, N18.3x, N18.4, N18.5, N18.6, N18.9, N19, N25.0, Z49.x, Z99.2 |
| Hypertension | H35.03x, I10, I11.9, I12.0, I12.9, I13.10, I13.11, I15.x, I16.x, I67.4, O10.01x, O10.02, O10.03, O10.11x, O10.12, O10.13, O10.21x, O10.22, O10.23, O10.31x, O10.32, O10.33, O10.41x, O10.42, O10.43, O10.91x, O10.92, O10.93, O11.x, O16.x |
| Coagulation defects | D66, D68.0, D68.1, D68.2, D68.318, D68.59, D68.8, D68.9 |
| Peptic ulcer disease | K25.x, K26.x, K27.x, K28.x |
| Dementia | F01.x, F02.x, F03.x, F04, F05, F06.1, F06.8, G13.2, G13.8, G30.x, G31.0x, G31.1, G31.2, G91.4, G94, R41.81, R54 |
| Rheumatologic diseases | M05.x, M06.x, M31.5, M32.x, M33.x, M34.x, M35.1, M35.3, M36.0 |
| Hemiplegia or paraplegia | G04.1, G11.4, G80.0, G80.1, G80.2, G81.x, G82.x, G83.x |
| Malignancy | C0x.x, C1x.x, C2x.x, C30.x, C31.x, C32.x, C33.x, C34.x, C37, C38.x, C39.x, C40.x, C41.x, C43.x, C45.x, C46.x, C47.x, C48.x, C49.x, C50, C51-58.x, C60-63.x, C76.x, C80.1, C81.x, C82.x, C83.x, C84.x, C85.x, C88.x, C9x.x |
| Metastatic solid tumour | C77.x, C78.x, C79.x, C80.0, C80.2 |
| Acquired immunodeficiency syndrome | B20 |
| Obesity (BMI≥30 kg/m^2^) | E66.9, Z68.3x, Z68.41, Z68.42, Z68.43, Z68.44, Z68.45 |

ICD-10-CM: The International Classification of Diseases, Tenth Revision, Clinical Modification, BMI: body mass index

**eTable 4. Pattern of organ involvement in patients with systemic sarcoidosis (n=654).**

| Organ involved | n | % |
| --- | --- | --- |
| Lung | 425 | 65.0 |
| Lymph | <10 | N/A |
| Lymph & Lung | 26 | 4.0 |
| Skin | 15 | 2.3 |
| Cranial nerves | <10 | N/A |
| Polyarthritis | <10 | N/A |
| Hepatic | 180 | 27.5 |

**eTable 5. Differences between patients with readmission and those without readmission at first admission**

|  | Systemic | |  |  | Isolated | |  |
| --- | --- | --- | --- | --- | --- | --- | --- |
|  | w/o readmission | with readmission | *P-value |  | w/o readmission | with readmission | *P-value |
| Number, N (%) | 453 (70.0) | 194 (30.0) | - |  | 741 (74.6) | 253 (25.5) | - |
| Age, years | 56.0 (48.0-63.0) | 56.0 (50.0-66.0) | 0.13 |  | 57.0 (49.0-66.0) | 57.0 (48.0-63.0) | 0.25 |
| Male, N (%) | 259 (57.2) | 110 (56.7) | 0.91 |  | 426 (57.5) | 155 (61.3) | 0.29 |
| Charlson comorbidity index, points | 2.0 (1.0-4.0) | 3.0 (2.0-5.0) | <0.0001 |  | 2.0 (1.0-4.0) | 3.0 (2.0-5.0) | <0.0001 |
| Anti-coagulant therapy, N (%) | 104 (23.0) | 77 (39.7) | <0.0001 |  | 182 (24.6) | 97 (38.3) | <0.0001 |
| Length of stay, day | 4.0 (2.0-7.0) | 5.0 (3.0-10.0) | 0.0009 |  | 3.0 (2.0-6.0) | 5.0 (2.0-9.0) | <0.0001 |
| Cardiac arrest, N (%) | <10 (N/A) | <10 (N/A) | 0.63 |  | <10 (N/A) | <10 (N/A) | 0.58 |
| **CIED implantation, N (%)** |  |  |  |  |  |  |  |
| PPM | <20 (N/A) | <10 (N/A) | 0.25 |  | <20 (N/A) | <10 (N/A) | 0.37 |
| ICD | 53 (11.7) | 12 (6.2) | 0.03 |  | 84 (11.3) | 19 (7.5) | 0.09 |
| CRT-P | <10 (N/A) | <10 (N/A) | 0.51 |  | <10 (N/A) | <10 (N/A) | 0.19 |
| CRT-D | <30 (N/A) | <10 (N/A) | 0.56 |  | <50 (N/A) | <10 (N/A) | 0.09 |
| **Cardiac interventions, N (%)** |  |  |  |  |  |  |  |
| Catheter ablation | <20 (N/A) | <10 (N/A) | 0.40 |  | 43 (5.8) | 13 (5.1) | 0.69 |
| Cardiac transplant | <10 (N/A) | <10 (N/A) | 0.39 |  | 18 (2.4) | 15 (5.9) | 0.007 |
| **HAC, US$** |  |  |  |  |  |  |  |
| Net value | 52,822.0 (24,686.0-120,567.0) | 56,451.0 (26,813.0-125,349.0) | 0.36 |  | 54,495.0 (20,811.0-125,049.0) | 56.413.0 (22,759.0-149,840.0) | 0.35 |
| Adjusted value | 14,754.1 (7,407.9-30,081.7) | 15,111.0 (7,663.1-33,625.3) | 0.34 |  | 14,385.6 (6,809.5-34,991.7) | 14,519.1 (7,111.7-36,074.3) | 0.39 |
| **Comorbidities, N (%)** |  |  |  |  |  |  |  |
| Ventricular tachycardia | 125 (27.6) | 59 (30.4) | 0.47 |  | 277 (37.4) | 91 (36.0) | 0.69 |
| Ventricular fibrillation | <10 (N/A) | <10 (N/A) | 0.35 |  | <10 (N/A) | <10 (N/A) | 0.15 |
| Atrioventricular block | 94 (20.8) | 29 (15.0) | 0.09 |  | 167 (22.5) | 49 (19.4) | 0.29 |
| Sick sinus syndrome | <10 (N/A) | <10 (N/A) | 0.28 |  | <30 (N/A) | <10 (N/A) | 0.30 |
| Atrial fibrillation | 110 (24.3) | 67 (34.5) | 0.007 |  | 204 (27.5) | 83 (32.8) | 0.11 |
| Heart failure | 307 (67.8) | 160 (82.5) | <0.0001 |  | 531 (71.7) | 207 (818) | 0.001 |
| Cerebrovascular diseases | <20 (N/A) | <10 (N/A) | 0.91 |  | 23 (3.1) | 11 (4.4) | 0.35 |
| Chronic Pulmonary disease | 140 (30.9) | 59 (30.4) | 0.90 |  | 131 (17.7) | 48 (19.0) | 0.64 |
| Peripheral vascular diseases | 180 (39.7) | 95 (49.0) | 0.03 |  | 340 (45.9) | 124 (49.0) | 0.39 |
| Liver diseases | 23 (5.1) | 10 (5.2) | 0.97 |  | <40 (N/A) | <10 (N/A) | 0.53 |
| Diabetes | 116 (25.6) | 67 (34.5) | 0.02 |  | 171 (23.1) | 74 (29.3) | 0.049 |
| Renal diseases | 102 (22.5) | 69 (35.1) | 0.001 |  | 176 (23.8) | 87 (34.4) | 0.001 |
| Hypertension | 134 (29.6) | 53 (27.3) | 0.56 |  | 197 (26.6) | 57 (22.5) | 0.20 |
| Coagulation defects | <10 (N/A) | <10 (N/A) | 0.70 |  | <20 (N/A) | <10 (N/A) | 0.25 |
| Obesity | 145 (32.0) | 53 (27.3) | 0.24 |  | 185 (25.0) | 59 (23.3) | 0.60 |
| **Hospital bed size, N (%)** |  |  |  |  |  |  |  |
| Small | <30 (N/A) | <10 (N/A) |  |  | 34 (10.4) | 10 (9.4) |  |
| Medium | <30 (N/A) | <10 (N/A) |  |  | 62 (18.9) | 20 (18.7) |  |
| Large | 130 (73.0) | 56 (81.2) | 0.36 |  | 232 (70.7) | 77 (72.0) | 0.95 |
| **Primary payer, N (%)** |  |  |  |  |  |  |  |
| Medicare/Medicaid | 238 (52.5) | 120 (61.9) |  |  | 346 (46.8) | 129 (51.0) |  |
| Private insurance | 195 (43.1) | 64 (33.0) |  |  | <400 (N/A) | <200 (N/A) |  |
| Others | 20 (4.4) | 10 (5.2) | 0.06 |  | <40 (N/A) | <10 (N/A) | 0.29 |

***HAC:*** *healthcare associated costs;* ***CIED****: cardiac implantable electronic devices;* ***PPM****: permanent pacemaker;* ***CRT-P****: cardiac resynchronization therapy with pacemaker;* ***CRT-D****: cardiac resynchronization therapy with defibrillator*

**eTable 6. Predictors of increasing LOS, HAC, and in-hospital death at readmission**

|  | LOS | | |  | | HAC (Adjusted) | |  | In-hospital death | |
| --- | --- | --- | --- | --- | --- | --- | --- | --- | --- | --- |
|  | β | P-value |  | | β | | P-value |  | OR (95% CI) | P-value |
| Systemic sarcoidosis | -0.03 | 0.48 |  | | -0.06 | | 0.13 |  | 0.89 (0.18-4.64) | 0.89 |
| Age | -0.0004 | 0.99 |  | | -0.02 | | 0.64 |  | 1.01 (0.93-1.09) | 0.89 |
| Male | 0.03 | 0.45 |  | | 0.007 | | 0.86 |  | 1.51 (0.27-8.30) | 0.64 |
| Charlson comorbidity index | 0.19 | 0.08 |  | | 0.17 | | 0.11 |  | 1.03 (0.43-2.47) | 0.95 |
| Anti-coagulant therapy | -0.06 | 0.14 |  | | -0.05 | | 0.27 |  | 1.92 (0.30-12.13) | 0.49 |
| Cardiac arrest | - | - |  | | - | | - |  | - | - |
| **CIED implantation** |  |  |  | |  | |  |  |  |  |
| PPM | -0.02 | 0.56 |  | | 0.003 | | 0.94 |  | - | - |
| ICD | -0.02 | 0.61 |  | | 0.07 | | 0.11 |  | - | - |
| CRT-P | - | - |  | | - | | - |  | - | - |
| CRT-D | 0.008 | 083 |  | | 0.08 | | 0.04 |  | - | - |
| **Cardiac interventions** |  |  |  | |  | |  |  |  |  |
| Catheter ablation | 0.02 | 0.64 |  | | 0.06 | | 0.14 |  | 8.97 (0.34-238.53) | 0.19 |
| Cardiac transplant | 0.18 | <0.0001 |  | | 0.30 | | <0.0001 |  | - | - |
| **Comorbidities** |  |  |  | |  | |  |  |  |  |
| Ventricular tachycardia | 0.07 | 0.09 |  | | 0.05 | | 0.24 |  | 1.67 (0.23-12.03) | 0..61 |
| Ventricular fibrillation | - | - |  | | - | | - |  | - | - |
| Atrioventricular block | -0.01 | 0.78 |  | | 0.02 | | 0.60 |  | 0.40 (0.02-7.48) | 0.54 |
| Sick sinus syndrome | 0.02 | 0.68 |  | | 0.01 | | 0.75 |  | - | - |
| Atrial fibrillation | -0.03 | 0.43 |  | | -0.03 | | 0.40 |  | 0.78 (0.13-4.74) | 0.54 |
| Heart failure | 0.03 | 0.51 |  | | -0.01 | | 0.84 |  | 2.29 (0.16-32.22) | 0.54 |
| Cerebrovascular diseases | -0.06 | 0.14 |  | | -0.03 | | 0.46 |  | 4.18 (0.18-98.34) | 0.37 |
| Chronic Pulmonary disease | -0.006 | 0.89 |  | | -0.06 | | 0.15 |  | 0.28 (0.02-3.26) | 0.31 |
| Peripheral vascular diseases | -0.15 | 0.001 |  | | -0.07 | | 0.11 |  | 0.20 (0.03-1.48) | 0.12 |
| Liver diseases | -0.002 | 0.96 |  | | -0.01 | | 0.74 |  | 1.58 (0.10-25.08) | 0.75 |
| Diabetes | -0.12 | 0.12 |  | | -0.14 | | 0.07 |  | 1.84 (0.08-40.10) | 0.70 |
| Renal diseases | 0.02 | 0.74 |  | | -0.03 | | 0.59 |  | 3.06 (0.26-36.06) | 0.37 |
| Hypertension | 0.05 | 0.24 |  | | 0.05 | | 0.24 |  | 2.34 (0.37-14.71) | 0.37 |
| Coagulation defects | -0.02 | 0.62 |  | | -0.07 | | 0.09 |  | - | - |
| Obesity | -0.02 | 0.59 |  | | -0.04 | | 0.32 |  | 2.99 (0.58-15.43) | 0.19 |
| **Hospital bed size** |  |  |  | |  | |  |  |  |  |
| Small | (ref.) | - |  | | (ref.) | | - |  | (ref.) | - |
| Medium | 0.02 | 0.71 |  | | -0.03 | | 0.64 |  | 0.91 (0.08-10.79) | 0.94 |
| Large | -0.009 | 0.87 |  | | 0.0005 | | 0.99 |  | 0.35 (0.04-2.96) | 0.33 |
| **Primary payer** |  |  |  | |  | |  |  |  |  |
| Medicare/Medicaid | (ref.) | - |  | | (ref.) | | - |  | (ref.) | - |
| Private insurance | -0.02 | 0.68 |  | | 0.007 | | 0.87 |  | 0.84 (0.14-5.19) | 0.85 |
| Others | -0.04 | 0.37 |  | | -0.04 | | 0.31 |  | - | - |

***LOS****: length of stay;* ***HAC****: healthcare-associated costs;* ***OR****: odds ratio;* ***CI****: confidence interval;* ***CIED****: cardiac implantable electronic devices;* ***PPM****: permanent pacemaker;* ***CRT-P****: cardiac resynchronization therapy with pacemaker;* ***CRT-D****: cardiac resynchronization therapy with defibrillator*
